# Evaluation of Polyphenolic Content, Antioxidant and Diuretic Activities of Six *Fumaria* Species

**DOI:** 10.3390/molecules22040639

**Published:** 2017-04-15

**Authors:** Ramona Păltinean, Andrei Mocan, Laurian Vlase, Ana-Maria Gheldiu, Gianina Crișan, Irina Ielciu, Oliviu Voștinaru, Ovidiu Crișan

**Affiliations:** 1 Department of Pharmaceutical Botany, “Iuliu Hațieganu” University of Medicine and Pharmacy, 400337 Cluj-Napoca, Romania; rpaltinean@umfcluj.ro (R.P.); mocan.andrei@umfcluj.ro (A.M.); gcrisan@umfcluj.ro (G.C.); irina.ielciu@umfcluj.ro (I.I.); 2 ICHAT and Institute for Life Sciences, University of Agricultural Sciences and Veterinary Medicine, Calea Mănăştur 3-5, 400372 Cluj-Napoca, Romania; 3 Department of Pharmaceutical Technology and Biopharmaceutics, “Iuliu Hațieganu” University of Medicine and Pharmacy, 400010 Cluj-Napoca, Romania; gheldiu.ana@umfcluj.ro; 4 Department of Pharmacology, Physiology and Physiopathology, “Iuliu Hațieganu” University of Medicine and Pharmacy, 400349 Cluj-Napoca, Romania; oliviu_vostinaru@yahoo.com; 5 Department of Organic Chemistry, “Iuliu Hațieganu” University of Medicine and Pharmacy, 400010 Cluj-Napoca, Romania; ovicrisan@yahoo.com

**Keywords:** *Fumaria* genus, LC-MS, polyphenols, antioxidants, diuretic activity

## Abstract

Romanian traditional medicine describes the use of aerial parts of *Fumaria* species to treat hepatobiliary diseases as well as diuretic agents. The present study aims to investigate the chemical composition, antioxidant properties, and diuretic effects of several *Fumaria* species. LC/MS analysis revealed that *Fumaria* species contain phenolic acids and high amounts of flavonoids with rutin and isoquercitrin as main compounds. Concerning antioxidant capacity, the most significant results were obtained for *F. capreolata* and *F. vailantii*. Both species showed a good correlation between the antioxidant capacity and a high amount of flavonoids. Furthermore, the extracts of *F. officinalis* and *F. schleicheri* produced a strong increase in urinary volumetric excretion of saline-loaded rats, 24 h after the oral administration of a single dose of 250 mg/kg bw. Moreover, both extracts of *F. officinalis* and *F. schleicheri* increased the urinary excretion of Na^+^ and K^+^. Results from the present study offer a new perspective concerning the chemical composition and bioactivities of traditionally used fumitory species.

## 1. Introduction

*Fumaria* genus (Papaveraceae family) comprises about 60 species [1] and is widely spread throughout the entire European continent, being found especially in the Mediterranean region, and Eastern and Western Europe [2]. The *Fumaria* species present a high risk of confusion due to their similar characters [3]. Identification of the species belonging to this genus is based on several specific morphological characters: presence or absence of the sepals, their length, shape of the fruit, length of the fruit pedicel, and length of the fruit pedicel bracteole [3,4]. Various species have been reported for their use in traditional medicine for the treatment of hepatobiliary pathologies [5]. Other sources cite the antifungal [6], antibacterial [7], and anti-inflammatory [8] activities of these species. These activities are especially due to the isoquinolinic alkaloids, among which protopine is the most frequently found [9]. Besides the isoquinolinic alkaloids in their composition, *Fumaria* species have proved to be important sources of polyphenols and flavonoids [10], which assign them antioxidant [11], diuretic and antiprotozoal [12] activity.

**Figure 1:**
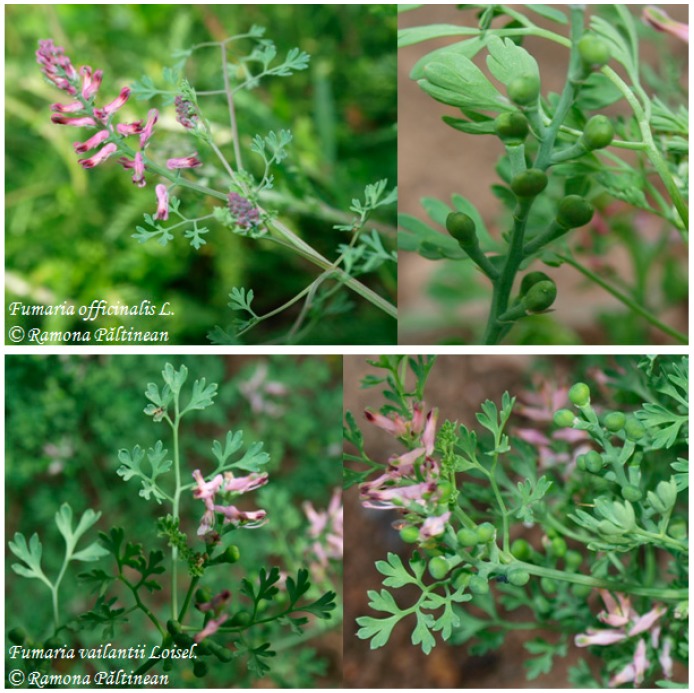
*Fumaria officinalis* L. and *Fumaria vailantii* Loisel.

Among the species that have proven important bioactivities, there is *Fumaria capreolata* L. [13] a species mostly found in the Western part of Europe [2]. The antioxidant activity of this species appears to be due to its composition in alkaloids [14]. Other biological activities reported for this species are anticholinesterasic and analgesic activities [15,16]. An important number of biological activities are associated and assessed to the *Fumaria officinalis* L. (Figure 1) [17,18], a species that is also spontaneous in the Western part of Europe [2]. The antibacterial [7] and the antioxidant [19] activities are the most frequently cited by scientific literature. Compounds that are responsible for these activities are mostly isoquinolinic alkaloids, but there are also authors that state the involvement of the polyphenols in some of these activities [11]. Other species that have proven significant biological activities can also be found in the Eastern part of Europe: *Fumaria parviflora* Lam. [20], *Fumaria vaillantii* Loisel. (Figure 1) [21,22], and *Fumaria rostellata* Knaf. [23]. The most frequently cited activities for these species are the antioxidant ones while several studies describe the antidiarrheal, antispasmodic, and bronchodilator activities [24]. Compounds that are responsible for these activities are polyphenols and flavonoids, found in important amounts especially in the aerial parts of these species [25,26].

*Fumaria schleicheri* Soyer-Willemet and *Fumaria jankae* = *Fumaria rostellata* × *Fumaria schleicheri* are two species mostly found in Central Europe [4]. More specifically, *Fumaria jankae* is an endemic species, which is reported to be found only in the Western part of Romania, in Bihor County, near Săcuieni [2,4]. Besides, *F. schleicheri* appears to be the most frequent *Fumaria* species in the Romanian country [4]. The existing scientific reports cite anticonvulsant activity, especially for *F. schleicheri* Soy.-Will. [27,28]. No scientific data about polyphenols exists on *F. jankae* to date, as it is an endemic species, present only in a specific geographic region of Romania.

In recent years, an important increase in the attempts to find natural sources of molecules with biological potential has been noticed [11]. Polyphenols from plants are such molecules, which have proven important antioxidant activity [21]. Antioxidants are important agents involved in the protection against oxidative stress that have proven to be one of the most important causes of many diseases nowadays [13]. Therefore, in recent years, many researchers have focused in this direction, with important results in the curative or adjuvant treatment of some diseases [11]. Several medicinal plants have been proven to contain significant amounts of polyphenols, which added an important value to their use in the therapy of numerous diseases [21]. This is the main reason why characterization and testing the biological activity of extracts obtained from these plants is essential for their introduction in therapy as phytopharmaceuticals [11].

There is little evidence of the antioxidant activity attributed to *Fumaria* species [11,13,14,20,21,22]. In this regard, and given the importance of antioxidants nowadays, the present study aims not only to increase the scientific evidence on the characterization of bioactive compounds and bioactivities of *Fumaria* species, but also to establish a connection between various species of the genus, found in the spontaneous flora of Europe. The species involved in the present work are: *F. officinalis* L., *F. capreolata* L., *F. rostellata* Knaf., *F. vaillantii* Loisel., *F. schleicheri* Soy.-Will, and *F. jankae* Hausskn. The originality of this study consists in the fact that it is the first report concerning the antioxidant activity of some *Fumaria* species found in the Romanian flora and especially of *F. jankae*, an endemic species. Moreover, in order to increase the number of reasons for the possible introduction of the extracts belonging to *Fumaria* species in phytotherapy, the present study also evaluated the diuretic activity of fumitory species, as they contain important amounts of polyphenols in their composition. The diuretic activity of *Fumaria* species is a subject less approached until present by the scientific literature [29]. In this context, the present study appears as the first approach of this type dealing with the quantification of bioactive compounds and the pharmacological evaluation of the Romanian species *F. schleicheri* and *F. jankae*.

The present work provides scientific evidence that *Fumaria* species represent an important and valuable source of polyphenols, which can lead to the potential development of natural antioxidants and diuretics. Further investigations may lead to the introduction in the pharmaceutical and food industry of some new antioxidant and diuretic molecules, which could be valuable in a variety of pathologies caused by oxidative stress.

## 2. Results and Discussion

### 2.1. HPLC Analysis of Polyphenols

The HPLC technique coupled with the MS detection is an analytical method used more and more often nowadays due to the fact that it provides important information about the analytes and it is, at the same time, a highly sensitive method [30]. An LC-MS method for the analysis of polyphenols in herbal materials was developed, and allowed the identification of several polyphenolic compounds by a single pass of the extract on a reverse-phase analytical column. Among the targeted compounds, only seven could be determined in aerial parts of six *Fumaria* species (*F. jankae* Hausskn, *F. vaillantii* Loisel., *F. schleicheri* Soy.-Will., *F. officinalis* L., *F. rostellata* Knaf., and *F. capreolata* L.). This method has been successfully tested previously [31] and has proven large applicability and good results. It allowed to simultaneous analysis of different classes of polyphenols by a single pass of the plant extract along the column, in 35 min. The results obtained for the polyphenol quantification of the analyzed extracts are presented in Table 1, in the order of their retention times. The external standard method was used in order to obtain the polyphenolic compounds content, expressed as μg compound/g dry weight (d.w.) of herbal material.

The compounds that were the most frequently found in the ethanolic extracts of all species were the quercetin glycosides, namely isoquercitrin, rutin, and quercitrin. Quantitatively, isoquercitrin was found in the largest amount in *F. capreolata* (2218.01 μg/g d.w.), while rutin in *F. vailantii* (2404.2 μg/g d.w.) and quercitrin in *F. capreolata* (250.84 μg/g d.w.). The presence of rutin as a major compound for the species *F. vailantii* was previously confirmed in the study performed by Orhan et al. [22]. Another class of compounds that was found in the chemical composition of all species were the flavonols, especially quercetin, found in the highest amount in *F. rostellata* (457 μg/g d.w.) and kaempferol, found in the highest amount in *F. officinalis* (44.14 μg/g d.w.). Less represented in the composition of the analyzed *Fumaria* species were the phenolic acids, among which ferulic acid was quantified in the composition of almost all species, except *F. vailantii* and in the highest amount was found in *F. rostellata* (36.42 μg/g d.w.). *p*-Coumaric acid could only be quantified in *F. rostellata*, *F. jankae*, and *F. officinalis*, the latter being also the one with the highest concentration, (113.54 μg/g d.w.) *F. officinalis*, *F. rostellata*, and *F. jankae* are the ones that, due to the fact that the amount of *p*-coumaric acid could be quantified, proved to be the species that was the richest in the variety of compounds. These findings confirm data existing in the scientific literature [11] that state the high amount of quercetin, *p*-coumaric acid, and ferulic acids, especially in the case of *Fumaria officinalis*.

The originality of the present study consists in being the first one that describes the polyphenolic composition of Romanian *F. schleicheri* and *F. jankae*. *F. schleicheri* turned out to be the species with the most important quantity of quercitrin (134.44 μg/g d.w.), but in its composition isoquercitrin (768.3 μg/g d.w.) and rutin (480.3 μg/g d.w.) were also found in significant amounts. Although in lowest amounts, the species had also been proven to contain quercetin (81.66 μg/g d.w.), ferulic acid (14.18 μg/g d.w.) and kaempferol (10.98 μg/g d.w.). *F. jankae*, an endemic species in Romania, is one of the tested species containing the greatest variety of compounds, of which the most important amounts were of isoquercitrin (173.4 μg/g d.w.), rutin (156.6 μg/g d.w.) and quercetin (103.68 μg/g d.w.). *p*-Coumaric acid could be detected and quantified in the content of this species (18.44 μg/g d.w.), together with ferulic acid (20.2 μg/g d.w.), quercitrin (52.16 μg/g d.w.), and kaempferol (13.62 μg/g d.w.), in lowest concentrations. This is the first report of the quantification of these compounds in the composition of *F. jankae*.

### 2.2. Determination of Phenolic Compounds Content

**Figure 2:**
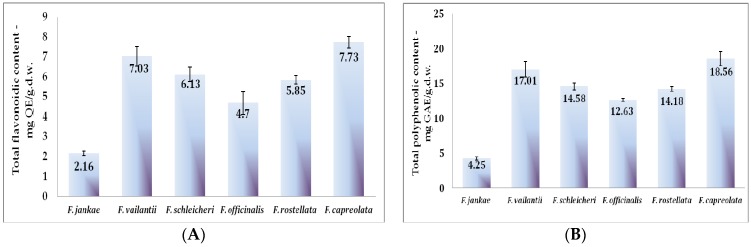

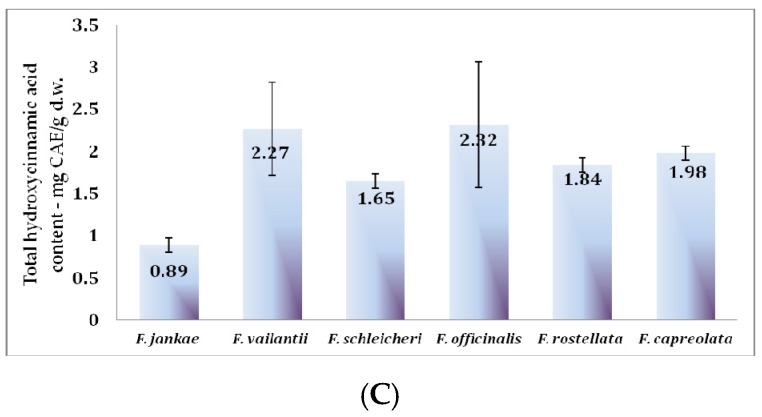
Total phenolic content (TPC) (**A**); total flavonoidic content (TFC) (**B**); and hydroxycinnamic acids (THC) (**C**) of the six *Fumaria* species.

Besides the determined compounds that could specifically be identified and quantified, many other polyphenolic compounds are widely distributed in aerial parts of *Fumaria* species and contribute to their overall antioxidant activity. The results of the amount of total polyphenolic contents (TPC), flavonoids (TFC), and hydroxycinnamic acids (THC) in the six analyzed species are represented in Figure 2. Thus, the TPC values were expressed as gallic acid equivalents (mg GAE/g d.w.). The quantification of the total flavonoidic content was carried out using the calibration curve of quercetin and presented as quercetin equivalents (mg QE/g d.w.) and the hydroxycinnamic acids content were expressed as caffeic acid equivalents (mg CAE/g d.w.).

The extract of *F. capreolata* extract contains the highest amount of total phenolics (18.56 mg GAE/g d.w.), flavonoidic compounds (7.73 mg QE/g d.w.) but lower values of hydroxycinnamic acids in comparison with *F. officinalis* and *F. vailantii*. Among the five analyzed species, the endemic taxon *F. jankae* presented the lowest amounts in terms of total phenolics, flavonoids, and hydroxycinnamic acids (4.25 mg/g d.w., 2.16 mg/g d.w., and 0.89 mg/g d.w., respectively).

Obviously, flavonoids are the major class of phenolic compounds for all the analyzed species. In a comparative study of five *Fumaria* species from Bulgaria led by Ivanov et al., *F. officinalis* presented the highest value in terms of total phenolic compounds (30.03 mg GAE/g d.w.) and total flavonoids (15.70 mg QE/g d.w.), while *F. rostellata* had values of 23 mg GAE/g d.w. and 14.01 mg QE/g d.w. [11]. Significantly lower values were registered by Riaz et al. who reported a value of 123.23 μg GAE/g d.w., for an ethyl acetate fraction of *F. indica* [20]. Nevertheless, no results were found for comparison regarding the endemic taxon of *F. jankae*. It is, therefore, the first report of the quantification of phenolic compounds content for this species.

### 2.3. Antioxidant Activity Assays

The antioxidant activity of herbal extracts, resulting from many compounds such as phenolic compounds and pigments, offers protective potential against oxidative stress in the human body. Thus, nowadays the trend is directed towards herbal supplements or functional foods with more phytochemicals and rich in natural antioxidants. In this part of the investigation, the goal was to create a comparative overview of the antioxidant activity of *F. jankae*, *F. vailantii*, *F. schleicheri*, *F. officinalis*, *F. rostellata*, and *F. capreolata*. As several authors suggest, the antioxidant capacity of herbal extracts and natural products cannot be evaluated by using a single test [32,33,34].

**Figure 3:**
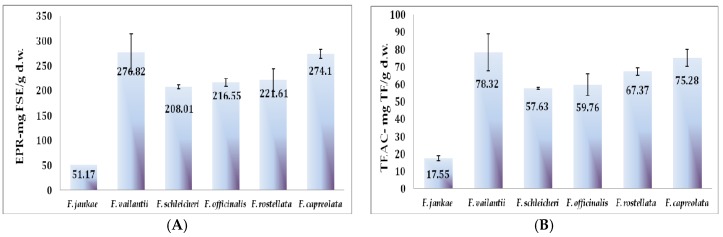
Antioxidant parameters by TEAC (**A**) and EPR spectroscopy of the six *Fumaria* species (**B**).

**Figure 4:**
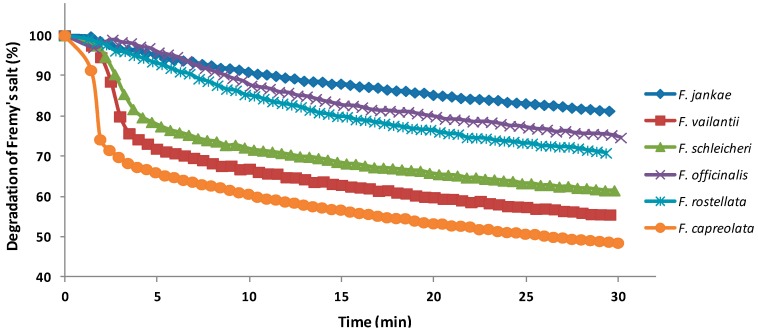
Degradation kinetics of the free radical Fremy’s salt by the six *Fumaria* species.

In this study, the widely-used TEAC assay was applied for characterizing the antioxidant capacity of the six *Fumaria* species (Figure 3A). Additionally, the antioxidants in fumitory 70% ethanolic extracts were assessed using electron spin resonance (EPR) spectrometry to evaluate their efficiency to reduce a synthetic free radical species, i.e., the semi-stable nitroxide radical Fremy’s salt (potassium nitrosodisulfonate) (Figure 3B and Figure 4).

The TEAC assay was carried out for all *Fumaria* species. *F. officinalis* (59.76 mg TE/g d.w.) and *F. schleicheri* (57.6 mg TE/g d.w.) exhibited similar antioxidant capacities and lower values in comparison to *F. vailantii* (78.32 mg TE/g d.w.), *F. capreolata* (75.28 mg TE/g d.w.), and *F. rostellata* (67.37 mg TE/g d.w.). Nonetheless, the lowest results in term of TEAC were registered for *F. jankae*, presenting thus a similar trend with the total phenolic and flavonoids content.

Data about antioxidant activity of *Fumaria* species is quite limited. However, in a report of Wasu et al. [19], *F. officinalis* ethanolic extract was able to inhibit lipid peroxidation. The study of Ivanov et al. presents the extract of *F. officinalis* as having the highest TEAC value (131.14 mmol TE), while *F. rostellata* only has 108.30 mmol TE.

The degraded amount of Fremy’s salt after 30 min of incubation time ranged between 276.82 mg FSE/g d.w. for *F. vailantii* and 51.17 mg FSE/g d.w. for *F. jankae* indicating a 45.4% of degradation for the first and just a 19.1% for the latter. Nevertheless, both assays presented a similar trend, *F. vailantii* and *F. capreolata* presenting the highest antioxidant capacity. Information about the antioxidant activity of *Fumaria* species using the electron paramagnetic resonance (EPR) spectrometry assay is not available, so far. Therefore, a comparison with data from other researchers is missing. The antioxidant activity of all analyzed *Fumaria* species evaluated by TEAC assay and EPR assay was also in line with their corresponding total phenolic content, indicating *F. vailantii* and *F. capreolata* as superior sources of antioxidant compounds.

### 2.4. Evaluation of Diuretic Activity

Diuretics drugs modulate the volume and composition of body fluids and are indicated in different clinical conditions like hypertension, heart failure, nephritic syndrome, and cirrhosis. Herbal diuretics produce very little toxicity and are considered safe and cost effective alternatives to synthetic drugs [35]. Nonetheless, many of the herbs used in traditional medicine have yet to be scientifically evaluated for their effectiveness and safety in relation with their indications. Up to date, information concerning the diuretic effects of *Fumaria* species is scarce [29,36]. Consequently, it is mandatory to bring new data regarding the influence of these medicinal plants into the diuresis process. In order to assess the diuretic activity, two species (*F. officinalis* and *F. schleicheri*) were analyzed, due to the fact that *F. officinalis* is the only fumitory species having a monograph in the European Pharmacopoeia and *F. schleicheri* is one of the most widespread species from Romania, which makes it a very easy and accessible raw plant material source for developing phytopharmaceuticals.

**Figure 5:**
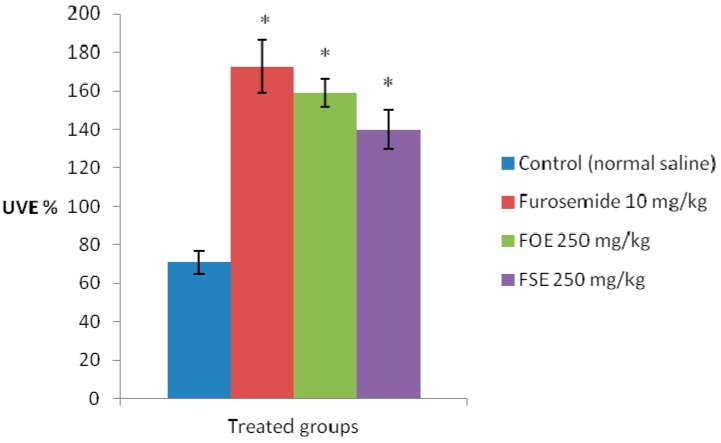
Effect of the extracts from *F. officinalis* (FOE) and *F. schleicheri* (FSE) on urinary volumetric excretion (UVE %) in saline-loaded Crl:WI rats, 24 h after the administration (* *p* < 0.05 vs. control).

As shown in Figure 5, the extracts from *F. officinalis* (FOE) and *F. schleicheri* (FSE) produced a strong increase in urinary volumetric excretion (UVE%) of saline-loaded Crl:WI rats, 24 h after the oral administration of a single dose of 250 mg/kg bw. Although the results were inferior to furosemide, the reference diuretic drug, they were statistically significant for both extracts (*p* < 0.05 vs. control).

Concerning the electrolyte excretion, both extracts from *F. officinalis* (FOE) and *F. schleicheri* (FSE) increased the urinary excretion of Na^+^ and K^+^ ions (U_Na_V) and (U_K_V), as shown in Table 2.

The administration of furosemide (10 mg/kg bw), the reference diuretic, led to a significant increase in electrolyte excretion (4.01 ± 0.35 mEq/kg/24 h Na^+^ and 4.71 ± 0.41 mEq/kg/24 h K^+^). The administration of FSE produced a higher electrolyte excretion than FOE, in both cases K^+^ excretion being superior even to furosemide (Table 2). The calculated Na^+^/K^+^ ratio for FOE and FSE treated groups did not show values above 10 at any moment of determination, thus indicating a lack of a potassium-sparing effect, situation similar with furosemide. On the contrary, both extracts from *Fumaria* produced a strong kaliuretic effect.

Our results showed a significant diuretic effect associated with an increase in urinary excretion of electrolytes in saline-loaded Crl:WI rats treated orally with the extracts from *F. officinalis* (FOE) and *F. schleicheri* (FSE). The pattern of the results suggested a tubular effect with a decrease in water and electrolyte reabsorption, further research being necessary in order to elucidate the mechanism of action and to evaluate the therapeutically importance of these findings. Moreover, the obtained results might be ascribed to the high content of flavonoids from these two species, as revealed by LC-MS findings. Additionally, several publications indicate that flavonoids-rich extracts are well-known herbal diuretic agents having the ability to modulate different physiological processes associated with diuresis [37,38,39].

## 3. Experimental Section

### 3.1. Plant Material

The plant material that was used for the analysis in this study consisted in the aerial parts of the six *Fumaria* species (*Fumariae herba*, as described by the European Pharmacopoeia, ninth edition) [40]. From the Romanian flora, five species were harvested in the Transylvania region: *Fumaria rostellata* Knaff*.* (Voucher No. 28.3.8.1.), *Fumaria vaillantii* Loisel. (Voucher No. 28.3.5.1.), *Fumaria schleicheri* Soy.Will. (Voucher No. 28.3.3.1.), and *Fumaria jankae* Hausskn. (Voucher No. 28.3.6.1.) One species was harvested from the spontaneous species of Belgium: *Fumaria officinalis* L. (Voucher No. 28.3.7.1.) and the species *Fumaria capreolata* L. (Voucher No. 28.3.10.1.) was harvested from the spontaneous flora of France. Identification of the taxa was made using the European Flora [2] and voucher specimens were deposited (in the Herbarium of the Department of Pharmaceutical Botany, Faculty of Pharmacy, Iuliu Hațieganu University of Medicine and Pharmacy, Cluj-Napoca, Romania.

### 3.2. Extraction Procedure

Before the extraction, the plant material was dried at room temperature and ground until it reached an appropriate degree of fineness. Powdered samples from the six species were extracted with ethanol 70% by sonication, for 30 min, using a plant material to solvent ratio of 1:20 (*w*/*v*). Afterwards, samples were centrifuged at 4500 rpm for 15 min and the supernatant was recovered and analyzed by the appropriate methods, according to each purpose of the study. In each case, the samples were analyzed in triplicate and the results were presented as mean ± SD.

### 3.3. Chemicals

The standards used for the LC-MS analysis (chlorogenic acid, *p*-coumaric acid, caffeic acid, rutin, apigenin, quercetin, isoquercitrin, quercitrin, hyperoside, kaempferol, myricetol, fisetin) were purchased from Sigma (St. Louis, MO, USA), whilst ferulic acid, sinapic acid, gentisic acid, gallic acid, patuletin, luteolin were purchased from Roth (Karlsruhe, Germany), and cichoric acid, caftaric acid from Dalton (Toronto, ON, Canada). HPLC grade methanol, ethanol, and analytical grade orthophosphoric acid, hydrochloric acid, and Folin-Ciocâlteu reagent were purchased from Merck (Darmstadt, Germany), ABTS (2,2′-azino-bis(3-ethylbenzothiazoline-6-sulfonic acid) diammonium salt; ≥98%), potassium peroxo-disulfate (≥99%), potassium nitrosodisulfonate (Fremy’s salt) (≥98%), trolox (6-hydroxy-2,5,7,8-tetramethylchromane-2-carboxylic acid; ≥97%) were purchased from Sigma Aldrich Chemie (Schnelldorf, Germany). Sodium carbonate, dipotassium hydrogen phosphate, potassium dihydrogen phosphate, and aluminium chloride were purchased as well from Merck (Darmstadt, Germany); gallic acid monohydrate (99.5%) was purchased from Serva (Heidelberg, Germany).

### 3.4. HPLC/MS Analysis

#### 3.4.1. Apparatus and Chromatographic Conditions

An Agilent Technologies 1100 HPLC Series system (Agilent, Santa Clara, CA, USA) equipped with G1322A degasser, G13311A binary gradient pump, column thermostat, G1313A autosampler and G1316A UV detector was used to carry out the chromatographic analysis of the *Fumaria* extracts. The HPLC system was coupled with an Agilent 1100 mass spectrometer (LC/MSD Ion Trap SL). Separation was performed on a reverse-phase analytical column (Zorbax SB-C18 100 × 3.0 mm i.d., 3.5 μm particles) and the temperature of the thermostat was set at 48 °C. The flow rate was 1 mL/min and the injection volume was 5 μL. The mobile phase was a binary gradient composed of methanol and acetic acid 0.1% (*v*/*v*). The elution was performed by a linear gradient beginning at 5% methanol, which ended with 42% methanol, for 35 min and then passed at 42% methanol for the next 3 min. Detection of the compounds was performed on both UV (at 330 nm until 17.5 min, then at 370 nm) and MS mode. The chromatographic data were processed using ChemStation and DataAnalysis software from Agilent.

The MS system was operated using an electro spray source of ions in a negative mode. The polyphenols were eluted in less than 40 min (Table 3) with these conditions. Each polyphenol was identified based on its specific mass spectra, which was recorded in a mass spectra library, after the analysis of a standard solution. The MS traces/spectra of the analyzed samples were compared to spectra from the library, which allowed the identification of compounds, based on spectral matching. Four polyphenols could not be quantified in the chromatographic conditions described above due overlapping (caftaric acid with gentisic acid and caffeic acid with chlorogenic acid). However, all four compounds can be selectively identified by MS detection (qualitative analysis) based on differences between their molecular mass and MS spectra.

MS signal was used only for qualitative analysis. For the quantification of the compounds identified by MS detection, the UV trace was used. The limit of quantification was 0.5 μg/mL, and the limit of detection was 0.1 μg/mL, for all compounds. The detection limits were calculated as minimal concentration producing a reproductive peak with a signal-to-noise ratio greater than three. Quantitative determinations were performed using an external standard method. Calibration curves in the 0.5–50 μg/mL range with good linearity (R^2^ > 0.999) for a five-point plot were used to determine the concentration of polyphenols in plant samples [41].

#### 3.4.2. Identification and Quantification of Polyphenols

The detection and quantification of polyphenols was performed in UV assisted by mass spectrometry detection. Due to peak overlapping, four hydroxycinnamic acids (caftaric, gentisic, caffeic, chlorogenic) were determined only based on MS spectra, whereas for the rest of the compounds the linearity of the calibration curves was very good (R^2^ > 0.998), with detection limits in the range of 18 to 92 ng/mL. The detection limits were calculated as the minimal concentration yielding a reproducible peak with a signal-to-noise ratio greater than three. Quantitative determinations were performed using an external standard method; retention times were determined with a standard deviation ranging from 0.04 to 0.19 min (Table 3). For all compounds, the accuracy was between 94.13% and 105.3%. Accuracy was checked by spiking samples with a solution containing each polyphenol in a 10 μg·mL^−1^ concentration. In all analyzed samples, the compounds were identified by a comparison of their retention times and recorded electrospray mass spectra with those of standards in the same chromatographic conditions.

### 3.5. Determination of Total Phenolic Content

The total phenolic content (TPC) of the extracts was measured using the Folin-Ciocâlteu method with some modifications. For a high-throughput of samples, a SPECTROstar Nano Multi-Detection Microplate Reader with 96-well plates (BMG Labtech, Ortenberg, Germany) was used. A mixture solution (the same solution which resulted after the extraction procedure) consisting of 25 μL sample extract, 125 μL of Folin-Ciocâlteu reagent, and an appropriate volume of sodium carbonate (Na_2_CO_3_) solution (7.5% *w*/*v*) was homogenized and incubated at room temperature in the dark for 2 h. The absorbance was measured at 760 nm. Gallic acid was used as a standard, and the content of TPC was expressed as gallic acid equivalents (GAE) in mg/g dry weight (dw) of herbal material [42,43].

### 3.6. Determination of Total Flavonoid Content

The total flavonoid content (TFC) was calculated and expressed as quercetin equivalents after the method described in the Romanian Pharmacopoeia (10th Edition). Each extract (5 mL) was mixed with sodium acetate (5 mL, 100 g/L), aluminum chloride (3 mL, 25 g/L), and made up to 25 mL in a calibrated flask with methanol. Each solution (the same solution which resulted after the extraction procedure, and no acidic hydrolysis was performed) was compared with the same mixture without reagent. The absorbance was measured at 430 nm. The total flavonoid content values were determined using an equation obtained from calibration curve of the quercetin graph (R^2^ = 0.999) [42,44].

### 3.7. Determination of Hydroxycinnamic Acids Content

The total hydroxycinnamic content (THC) was determined using the method from the European Pharmacopoeia, ninth edition, from the monograph *Fraxini folium* using caffeic acid instead of chlorogenic acid. The total content of hydroxycinnamic acids was determined by using a spectrophotometric method with Arnow’s reagent (10 g sodium nitrite and 10 g sodium molybdate made up to 100 mL with distilled water). The absorbance was measured at 525 nm. The percentage of hydroxycinnamic acids, expressed as caffeic acid equivalents on dry material plant (mg CAE/g plant material), was determined using an equation that was obtained from calibration curve of caffeic acid (R^2^ = 0.994) [40].

### 3.8. Antioxidant Assays

#### 3.8.1. ABTS assay using Trolox Equivalents (TEAC)

The effects of extract on the synthetic ABTS radical were estimated by the method previously described by Toma et al. 2015 with some modifications, and the results were expressed as Trolox equivalents (antioxidant capacity) [45,46].

#### 3.8.2. EPR Measurements

EPR measurements were carried out by the method previously described by Mocan et al. 2015 with some modifications concerning sample dilution [45,46]. Appropriate extract dilutions (1:400) were prepared and 25 μL aliquots were allowed to react for 30 min with an equal volume of a solution of Fremy’s salt (1 mM in phosphate buffer, pH 7.4). EPR spectra of Fremy’s radical were obtained with a Bruker Elexsys E500 spectrometer (Bruker, Billerica, MA, USA). The antioxidant activity expressed as mg Fremy’s salt equivalents reduced by 25 μL diluted extract was calculated by comparison with a control reaction with 25 μL Fremy’s salt and 25 μL of extraction solvent.

### 3.9. Evaluation of Diuretic Activity

For the in vivo pharmacological studies, the extracts from *F. officinalis* (FOE) and *F. schleicheri* (FSE) were spray-dried and suspended in a mixture of Tween 80 and normal saline solution (1:100 *v*/*v*).

#### 3.9.1. Animals

Four groups of male Charles River Wistar (Crl:WI) rats (*n* = 6) with a medium weight of 150 g were obtained from the Practical Skills and Experimental Medicine Center of the “Iuliu Haţieganu” University of Medicine and Pharmacy Cluj-Napoca (Romania). The animals were housed in polycarbonate type IV-S open-top cages (Tecniplast, Italy) and maintained under standard conditions (22 ± 2 °C, a relative humidity of 45% ± 10%, 12:12-h light: dark cycle). The animals had access to a standard pelleted feed (Cantacuzino Institute, Bucharest, Romania) and filtered water ad libitum throughout the experiment, except for the day when the test substances were administered. All experimental protocols were approved by the Ethics Committee of the “Iuliu Hațieganu” University of Medicine and Pharmacy, Cluj-Napoca, Romania and were conducted in accordance with the EEC Directive 63/2010 which regulates the use of laboratory animals for scientific purposes.

#### 3.9.2. Diuretic Activity

For the evaluation of the diuretic activity of the extracts from *F. officinalis* (FOE) and *F. schleicheri* (FSE), a method using isotonic saline solution as hydrating fluid was applied (Kau et al. 1984) [47]. Thus, the negative control group of Crl:WI rats was treated by intragastric route with 25 mL/kg bw isotonic saline solution (Braun Gmbh, Marktheidenfeld, Germany) while the positive control group was treated by the same route with 10 mg/kg bw furosemide (Zentiva SA, Bucharest Romania), a reference diuretic drug, dissolved also in a volume of 25 mL/kg bw isotonic saline solution (Sadki et al. 2010) [48]. The other two groups of Crl:WI rats were treated also by intragastric route, with 250 mg/kg bw FOE and 250 mg/kg bw FSE, respectively, suspended in the same volume of 25 mL/kg bw isotonic saline solution.

Afterwards, the animals were individually placed in metabolic cages, the environmental temperature being maintained at 24 °C. The cumulative urine output was recorded for each animal, 24 h after the administration of a single dose from the tested substances. The urinary volumetric excretion (UVE %) was calculated with the formula:

UVE % = (Volume collected/Volume administered) × 100

Also, the urinary excretion of sodium and potassium ions (U_Na_V and U_K_V) was determined in the collected urine samples, 24 h after the substance administration, by a potentiometric method, using a VITROS 250 Chemistry System auto-analyzer (Johnson and Johnson Clinical Diagnostic), being expressed in mEq/kg. Finally, the ratio Na^+^/K^+^ was calculated based on the values of U_Na_V and U_K_V, in order to indicate a possible potassium-sparing effect [47,48,49,50].

### 3.10. Statistical Analysis

Data were expressed as mean values ± SD and were statistically analyzed by one way ANOVA method. The differences between the treated groups and the negative control group were evaluated by Dunnett’s *t*-test, with *p* values < 0.05 being considered statistically significant.

## 4. Conclusions

*Fumaria* species are used to treat hepato-biliary diseases as well as diuretic agents in traditional medicine. In this study the chemical composition, antioxidant properties and diuretic effects of several *Fumaria* species were investigated. LC/MS analysis revealed that *Fumaria* species contain phenolic acids and high amounts of flavonoids. Rutin and isoquercitrin were found as main compounds. As shown by the LC/MS analysis the highest amount of rutin was found in *F. vailantii*, whilst the highest amount of isoquercitrin was found in *F. capreolata*. The highest antioxidant capacity was obtained for *F. capreolata* and *F. vailantii*, showing as well a good correlation between the antioxidant assays and the high amount of flavonoids found in these two species. Additionally, the extracts from *F. officinalis* and *F. schleicheri* produced a strong increase in urinary volumetric excretion of saline-loaded rats, 24 h after the oral administration of a single dose of 250 mg/kg bw. Moreover, both extracts of *F. officinalis* and *F. schleicheri* increased the urinary excretion of Na^+^ and K^+^. Results from the present study offer a new perspective concerning the chemical composition and biological effects of traditionally-used fumitory species.

## Figures and Tables

**Table 1:** The polyphenolic compounds content in *Fumaria* extracts (μg/g d.w.).

Polyphenolic Compound	*Fumaria jankae*	*Fumaria vailantii*	*Fumaria schleicheri*	*Fumaria officinalis*	*Fumaria rostellata*	*Fumaria capreolata*
*p*-Coumaric acid	18.44 ± 0.1 *	NF	NF	113.54 ± 1.02 *	67.80 ± 0.8 *	NF
Ferulic acid	20.20 ± 0.2 *	NF	14.18 ± 0.13 *	25.30 ± 0.2 *	36.42 ± 0.5 *	16.23 ± 0.56 *
Isoquercitrin	173.40 ± 1.7 *	820.70 ± 8.5 *	768.30 ± 6.5 *	506.20 ± 4.5 *	1279.94 ± 10 *	2218.01 ± 9.8 *
Rutin	156.60 ± 1.65 *	2404.20 ± 20.8 *	480.30 ± 3.9 *	854.40 ± 7.8 *	121.06 ± 1.2 *	130.06 ± 1.52 *
Quercitrin	52.16 ± 0.6 *	119.48 ± 1.2 *	134.44 ± 1.4 *	48.40 ± 0.4 *	97 ± 1.02 *	250.84 ± 1.81 *
Quercetin	103.68 ± 1.2 *	146.62 ± 1.5 *	81.66 ± 0.9 *	348 ± 3.3 *	457 ± 3.69 *	55.31 ± 0.13 *
Kaempferol	13.62 ± 0.1 *	13.62 ± 0.16 *	10.98 ± 0.1 *	44.14 ± 0.3 *	30.80 ± 0.4 *	5.67 ± 0.02 *

Note: * = SD = standard deviation; NF = not found, below the limit of quantification.

**Table 2:** Effect of the extracts from *F. officinalis* (FOE) and *F. schleicheri* (FSE) on urinary excretion of sodium ions (U_Na_V) and potassium ions (U_K_V) in saline-loaded Crl:WI rats, 24 h after the administration.

Group	Dose	U_Na_V (mEq/kg/24 h)	U_K_V (mEq/kg/24 h)	Na^+^/K^+^ Ratio
Control	-	1.68 ± 0.15	2.40 ± 0.22	0.7
Furosemide	10 mg/kg	4.01 ± 0.35 *	4.71 ± 0.41 *	0.85
FOE	250 mg/kg	3.21 ± 0.16 *	5.82 ± 0.29 *	0.55
FSE	250 mg/kg	3.59 ± 0.29 *	6.08 ± 0.49 *	0.59

Note: * *p* < 0.05 vs. control; Values are expressed as mean ± SD.

**Table 3:** Retention times (RT) of the tested polyphenolic compounds (min).

No.	Phenolic Compounds	*m*/*z*	RT ± SD (min)
1.	Caftaric acid	311	3.54 ± 0.05
2.	Gentisic acid	179	3.52 ± 0.04
3.	Caffeic acid	179	5.60 ± 0.04
4.	Chlorogenic acid	353	5.62 ± 0.05
5.	*p*-Coumaric acid	163	9.48 ± 0.08
6.	Ferulic acid	193	12.8 ± 0.10
7.	Sinapic acid	223	15.00 ± 0.10
8.	Cichoric acid	473	15.96 ± 0.13
9.	Hyperoside	463	18.60 ± 0.12
10.	Isoquercitrin	463	19.60 ± 0.10
11.	Rutin	609	20.20 ± 0.15
12.	Myricetin	317	21.13 ± 0.12
13.	Fisetin	285	22.91 ± 0.15
14.	Quercitrin	447	23.64 ± 0.13
15.	Quercetin	301	26.80 ± 0.15
16.	Patuletin	331	29.41 ± 0.12
17.	Luteolin	285	29.10 ± 0.19
18.	Kaempferol	285	32.48 ± 0.17
19.	Apigenin	279	33.10 ± 0.15

Note: SD = standard deviation.
